# What shapes the range edge of a dominant African savanna tree, *Colophospermum mopane*? A demographic approach

**DOI:** 10.1002/ece3.7377

**Published:** 2021-03-29

**Authors:** Nicola Stevens

**Affiliations:** ^1^ Environmental Change Institute School of Geography and the Environment University of Oxford Oxford UK

**Keywords:** climate change, *Colophospermum mopane*, demography, range edge, savanna, species distribution limits

## Abstract

Climate is widely assumed to be the primary process that limits the distribution ranges of plants. Yet, savannas have vegetation not at equilibrium with climate, instead its structure and function are shaped by interactions between fire, herbivory, climate, and vegetation. I use the rich literature of a dominant African savanna woody plant, *Colophospermum mopane,* to demonstrate that climate and disturbance interact with each demographic stage to shape this species range limits. This synthesis highlights that climate‐based predictions for the range of *C. mopane* inadequately represents the processes that shape its distribution. Instead, seed bank depletion and rainfall limitation create a demographic bottleneck at the early seedling stage. The legacy of top‐kill from disturbance changes tree stand architecture causing a critical limitation in seed supply. Exposure to top‐kill at all demographic stages causes a vigorous resprouting response and shifts tree architecture from that of 1–2 stemmed tall trees to that of a short multi‐stemmed shrub. The shorter, multi‐stemmed shrubs are below the height threshold (4 m) at which they can produce seeds, resulting in shrub‐dominated landscapes that are effectively sterile. This effect is likely most pronounced at the range edge where top‐kill‐inducing disturbances increase in frequency. The proposed mechanistic, demographic‐based understanding of *C. mopane's* range limits highlights the complexity of processes that interact to shape its range edges. This insight serves as a conceptual model for understanding the determinants of range limits of other dominant woody savannas species living in disturbance limited ecosystems.

## Graphical abstract

Climate alone is unlikely to capture the processes that shape range edges in disturbance limited ecosystems. Here, we draw on the extensive information on a tropical savanna species, *Colophospermum mopane,* to demonstrate that disturbance and climate interact to shape this species range edge and the legacy of disturbance changes tree stand architecture causing a critical limitation in seed supply. Our proposed mechanistic understanding highlights the complexity of processes that interact to shape ranges and serves as a model for determining other dominant savanna trees range limits.

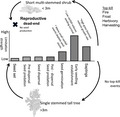

## 1 INTRODUCTION

Deciphering the determinants of species distribution ranges is a question that has intrigued ecologists for decades (Darwin, 1859; McArthur, 1972; Von Humboldt, 1817; Woodward & Williams, 1987). This issue has experienced a resurgence in interest due to large‐scale changes to climate and habitat (Gaston, 2009; Sexton et al., 2009). Climate is considered to be the primary determinant of species ranges (Sheldon, 2019; Woodward, 1987), but in disturbance‐driven systems, climate and disturbance can interact to shape species distribution ranges, and this is most likely for tropical savannas (Pausas & Bond, 2019). Tropical savannas are characterized by a grassy ground layer (generally dominated by C4 grasses) with a woody overstory varying from 0% to 80% cover (Ratnam et al., 2011). Savannas are the largest biome not at equilibrium with climate and are instead shaped by interactions between fire, climate, and vegetation (Bond et al., 2005; Lehmann et al., 2014; Staver et al., 2011) which in turn shape the ecology, evolution, and biogeography of plants (Bakker et al., 2016; Charles‐Dominique et al., 2017; Magadzire et al., 2019; Pausas & Bond, 2019; Stevens et al., 2018).

In this synthesis, I focus on the monodominant tropical savanna woody species *Colophospermum mopane* (mopane) (Kirk ex Benth.) Kirk ex J.Léon. Mopane is a dominant leguminous tree or multi‐stemmed shrub. It is widespread across the hot, low‐lying savannas of tropical southern Africa where it almost singly dominates ~ 35% of southern African savannas and has a strong tendency to form monospecific stands on nutrient‐rich and nutrient‐poor soils (Mapaure, 1994; Timberlake, 1995) (Figure 3). Despite the low compositional diversity in mopane stands, it is an ecologically and socio‐economically valuable species (Makhado et al., 2014; Ryan et al., 2016). Ecologically, mopane plays a significant role in the carbon cycle, hosts a unique biodiversity, and provides browse in times of drought. It provides valuable ecosystem services and is used extensively for fuelwood, building material, medicine and is the host of the economically important mopane caterpillar (the larvae of *Gonimbrasia belina*), a highly‐valued protein source (Bruschi et al., 2017; Makhado, Potgieter, et al., 2014; McNicol et al., 2018; Moura et al., 2017; Timberlake, 1995).

Mopanes’ sheer numerical dominance and value to rural livelihoods have made it a topic of research interest around some key themes. Mopane is a woody encroaching species where notable increases in its density within its range have been observed (O’Connor et al., 2014; Smit & Rethman, 1998a); a trend that can impact biodiversity and alter ecosystem service delivery. Secondly, the peculiar distribution of mopane has intrigued ecologists for decades, where after dominating many thousands of hectares it reaches its southernmost distribution limit along the Timbavati and Olifants Rivers in the Kruger National Park (South Africa) (Henning & White, 1974; Makhado et al., 2014; Mapaure, 1994). Remarkably, its distribution stops so abruptly that it is almost possible to step over the range edge! Thirdly, bioclimatic models predict that *C. mopane* will undergo extensive south‐ and westward range expansion as a result of climate change (Rutherford et al., 1999). Answers to these questions would be advanced by a clear mechanistic understanding of what limits the success or failure of mopane populations.

The majority of empirical approaches to identify factors causing range limits of mopane have relied on correlative climate studies (Burke, 2006; Henning & White, 1974; Makhado, Mapaure, et al., 2014; Okitsu, 2005; Rutherford et al., 1999; Stevens et al., 2014). These studies summarized and reanalyzed in a recent review (see Makhado, Mapaure, et al., 2014) conclude that a combination of high temperatures, low‐moderate rainfall (300 – 800 mm), and low altitudes (<800 m.a.s.l) are indicative of mopane's success. The presence of acidic soils, declining minimum winter temperature (< 5°C), and the prevalence of frost are generally the factors hypothesized to limit the distribution of mopane (Burke, 2006; Henning & White, 1974; Makhado, Mapaure, et al., 2014; Okitsu, 2005; Siebert, 2012; Stevens, Swemmer, et al., 2014; Timberlake, 1995). Yet, explicit experimental manipulations in the form of latitudinal and altitudinal transplant experiments demonstrate that climate alone does not limit the growth of this species (Stevens et al., 2018). In the absence of fire and herbivory, mopane survives and grows in the presence of frost, rainfall < 800 mm and at altitudes exceeding 800m.a.s.l. (Stevens et al., 2018). This highlights that we do not understand the mechanisms that culminate to limit or promote success of mopane. To address this, I draw on the rich literature on mopane to synthesize how interactions with disturbance at different demographic stages determine its distribution.

## 2 DEMOGRAPHIC CONSTRAINTS ON THE DISTRIBUTION OF *C. MOPANE*

I synthesize the information available about *C. mopane* at each life‐history stage (Figure 2) and highlight limitations and demographic hurdles which culminate in shaping the distribution of this species.

### 2.1 Seed stage

#### 2.1.1 Seed set limitation?

There is no evidence of low floral abundance or pollination success in limiting seed set in mopane. Despite irregularity in flowering, floral damage by insects and potential density‐dependent reduction in flower abundance an overall high seed and floral productivity suggests these impacts are not significant (Smit & Rethman 1998b). Secondly, mopane produces large amounts of pollen and the flowers are likely both wind and insect pollinated (Banks & Gasson, 2000; Jordaan et al., 2002; Krüger et al., 1999), therefore, given these two modes of pollination it is unlikely that lack of pollination opportunities limits seed set.

#### 2.1.2 Seed limitation

Seed production in mopane is limited by a clear height threshold (Caughley, 1976). Mopane trees can produce large crops of seed in excess of 10 000 seeds per tree (*pers obs*), but seed production is unlikely or extremely low in plants shorter than 4m in height (Caughley, 1976, Stevens *unpublished data*). Seed production is also impacted where trees growing with high rates of intra‐tree competition produce smaller and lighter seeds and pods; however, this does not translate into differences in germination rates (Smit & Rethman, 1998b). There is little quantitative information on seed predation, but limited data suggests postdispersal seed predation is low, especially when compared to other common African genera (Midgley & Bond, 2001), with invertebrate predation rates ranging between 20% in low grass cover areas and 23% with high grass cover, and vertebrate seed predation ranging from 9% in low grass areas to 11% in high grass areas. Both vertebrate and invertebrate seed predation was highest under the canopy of the parent plants (29% invertebrate predation and 12% vertebrate predation). Most seeds were attacked by seed borers from the genus *Araecerus* (Mlambo et al., 2007). Given the high rates of seed production and low rates of predation, there is unlikely a seed supply limitation when trees exceed 4m in height. In stands where mopane is short, seed production by multi‐stemmed shrubs will be severely limited and the landscape seed production will be a product of the number of trees exceeding 4m in the population (Caughley, 1976). Throughout the rest of the review, a multi‐stemmed plant shorter than 4m is referred to as a shrub.

#### 2.1.3 Seed dispersal limitation

Seed dispersal influences how many seeds make it to safe sites where they can receive the appropriate germination cues and conditions for seedling recruitment. The seed and pod of mopane are inseparable (Jordaan & Wessels, 1999) and thus together make up the dispersal unit (Jordaan & Krüger, 2005). Mopane pods are thin flattened indehiscent structures (Figure 2). The large surface area may facilitate wind dispersal and floating ability. Seed dispersal has traditionally been ascribed to epizoochory (i.e., adherence of seeds to the hooves of passing animals) due to the sticky and resinous nature of the pod (Jarman & Thomas, 1968). Wind and water are more likely dispersal agents (Styles & Skinner, 1997). Wind can move seeds 50–500 m (Styles & Skinner, 1997) and water run‐off during storms disperses seeds resulting in the aggregation of seeds in depressions after water levels have dropped (Mlambo & Nyathi, 2004). Endozoochorous dispersal (ingestion by mammals) is not possible as the large and fragile seeds cannot escape mastication in the herbivore's digestive tract (Styles & Skinner, 1997), a conclusion supported by the absence of mopane seeds and seedlings in herbivore dung (Dudley, 2000; Styles & Skinner, 1997). Potential alternative dispersal mechanisms such as the transportation of tree branches holding seeds by elephants, or scatter hoarding by squirrels or other rodents have not been investigated. Dispersal opportunities in mopane do not appear to be limiting, and low genetic variation between mopane populations ~ 400km apart confirm that neither pollen nor seed dispersal is limiting (Villoen et al., 2003).

#### 2.1.4 Seed germination limitation

Seed dormancy is absent in mopane seeds (Mlambo & Nyathi, 2004; Mlambo et al., 2007), and in field conditions, seeds are short‐lived and seldom remain viable for more than a year (Jordaan & Wessels, 1999; Mlambo & Nyathi, 2004); however, under laboratory conditions, seeds can remain viable for up to five years (Mojeremane & Lumbile, 2005). In a newly formed fruit, the degradation of the pericarp is initiated by endophytic fungi even before seeds are dropped which ultimately facilitates water penetration but reduces longevity in the field (Jordaan & Krüger, 2005; Jordaan et al., 2002; Mojeremane & Lumbile, 2005).

Mopane produces an indehiscent, 20–30 mm long, kidney ‐shaped fruit that contains a single seed (Jordaan & Wessels, 1999) (Figure 2). The seeds of mopane have a thin, undifferentiated seed coat, which is highly permeable to water (Jordaan & Krüger, 2005), and after 20 min exposure to moisture, seeds start to produce mucilage, which allows seeds to easily imbibe water and lower the evaporation of moisture from the seed (Jordaan & Krüger, 2005). This rapid uptake of water by the seeds is an adaptation for low and inconsistent rainfall events and facilitates high levels of germination after the seed receives water (Choinski Jr & Tuohy, 1991; Stevens et al., 2014).

Mopane germinates easily and reaches a high germination percentage and rate under a wide range of environmental conditions, achieving nearly 100% germination from 20^o^C to 40^o^C under the absence of water stress (Choinski Jr & Tuohy, 1991; Stevens, Seal, et al., 2014). Thermal time models suggest that under average summer growing season temperatures, 50% of germination can occur within 2 days when there is sufficient water (Stevens, Seal, et al., 2014). Mopane experiences enhanced germination success under moderate water stress when temperatures exceed 30°C (Stevens, Seal, et al., 2014). This represents a germination strategy that is likely to be adaptive in hot arid environments where regular rainfall events are infrequent. However, mopane's high germination rate in the presence of even low amounts of water may create a problem with seed supply, especially given a limited seed bank. If the initial germination event is not followed up by suitable rainfall, germination failure can result in rapid depletion of the seasons seed supply. Therefore, the arid‐adapted strategy of high germinability is best maintained when the number of rainfall events that promote germination is either large (facilitating successful establishment) or infrequent (reducing loss of seasons seed supply) (Stevens, Seal, et al., 2014).

### 2.2 Seedling establishment

Although seed germination is high, the establishment of the new seedlings is strongly limited by rainfall (Mlambo & Nyathi, 2004). Successful establishment of the newly germinated seed requires that the radicle grows sufficiently long to extend out of the soil evaporation zone into a deeper soil layer to where the soil moisture is more constant. Importantly, this needs to occur within a window period following a rainfall event where there is still sufficient soil moisture in the evaporation zone (Stevens, Seal, et al., 2014). Laboratory experiments demonstrate that mopane radicle extension is enhanced even under low water stress (−0.2 ‐ −0.8MPa), which significantly stimulates root elongation (Choinski Jr & Tuohy, 1991; Johnson et al., 1996; Stevens, Seal, et al., 2014). This arid adaptation facilitates a rapid elongation out of the “risky” evaporation zone and provides an essential survival trait to enhance early seedling establishment success in mopane. This adaptation is not present in co‐occurring *Vachellia* and *Senegalia* species (Choinski Jr & Tuohy, 1991). Modeling exercises indicate mopane seedlings require a window period of ~ 29 days of moist shallow soil for the radicle to extend to deeper soil layers where soil moisture is more consistent. The combination of conditions for successful early establishment occurs on average every 1 in 3 years in an arid savanna, making early seedling establishment a critically limiting stage and a likely cause of a demographic bottleneck.

#### 2.2.1 Grass impacts on seedling establishment

Seedling survival under tree canopies of both conspecifics and heterospecific species is near zero (Mlambo & Nyathi, 2004; Mlambo et al., 2007), but open areas with bare ground or sparse grass cover are favorable sites for seedling recruitment (Mlambo & Nyathi, 2004). Seedling survival in the presence of grass is reduced by 50% regardless of the amount of grass biomass (Mlambo et al., 2007; Stevens et al., 2018; Van Der Waal et al., 2009), most likely due to competition for soil moisture (Van Der Waal et al., 2009) and light (Mlambo et al., 2007). Additionally, in the presence of grass, seedling growth is significantly suppressed (Stevens et al., 2018; Vadigi & Ward, 2013; Van Der Waal et al., 2009) and seedlings are more vulnerable to wet‐season droughts where premature leaf senescence can occur (Van Der Waal et al., 2009). Although it is notable that in mopane stands grass biomass is low (Smit, 2001; Ward et al., 2013), with grass biomass showing a strong negative correlation with increasing *C. mopane* density (Vadigi & Ward, 2013; Van Der Waal et al., 2009). This pattern is hypothesized to be caused by allelopathic effects of mopane, although little evidence exists to support or refute this (Georginah & Maanda, 2015; Khavhagali & Ligavha‐Mbelengwa, 2009; Mlambo & Nyathi, 2004; Timberlake, 1995).

#### 2.2.2 Fire and herbivory impact on seedling establishment

Top‐kill from fire and herbivory does not significantly lower seedling survival but it does change the plant architecture. Mopane seedlings become herbivore proof from ~ 2 months old, when herbivory no longer causes mortality (Vadigi & Ward, 2014). Manipulative experiments on 6‐month‐old seedlings demonstrate that pruned seedlings experienced reductions in height, leaf area, and above‐ground biomass; however, the reductions were not proportionately as large as the amount of biomass removed, indicating that seedlings show some compensatory regrowth capacity. Architectural changes included multi‐stemmed regrowth, increased branching with a shorter lateral branch length and a reduction in total main stem length (Archibald et al *unpublished data,* Hartnett et al., 2012; Stevens, 2014). As seedling germination and establishment happens in the growing season and fire generally during the dry season, fire often impacts seedlings that are ~ 6 months old and results in a low mortality rate of 5% (Ben‐Shahar, 1996a). Observations on the impact of fire on seedlings indicate that they will still resprout in the following growing season (Mlambo & Nyathi, 2004) suggesting considerable carbohydrate storage in the roots.

Other potential causes of seedling mortality such as pathogens and herbivory by insects or rodents have been acknowledged (Mlambo & Nyathi, 2004) but not quantified. Experiments where seedlings have experienced “mopane caterpillar” type defoliation show that mopane seedlings are well adapted to complete defoliation with the subsequent regrowth causing a reduction in leaf size, but not leaf area, and an increase in the number of lateral branches and biomass (Hartnett et al., 2012). However, herbivory by the mopane caterpillar (*Gonimbrasia belina*) is limited in this stage as mopane caterpillars tend to not oviposit on trees less than 1m in height (Wiggins, 1997).

### 2.3 Sapling stage

I define a sapling as a seedling that has survived the dry season and enters the second growing season as a sapling (Wigley et al., 2020). Savanna tree saplings can resprout repeatedly following disturbance and may remain “trapped” within the sapling stage for decades resulting in a high variability in the age of saplings (Bond, 2008; Gignoux et al., 1997). The density of mopane saplings can be as high as 150 individuals ha^‐1^ (Smit & Rethman, 1998b). At this stage, the saplings are short and are vulnerable to top‐kill events such as frost (Whitecross et al., 2012), fire (Higgins et al., 2000; Kennedy & Potgieter, 2003), and herbivory (Styles & Skinner, 2000). Successful transition from this stage requires saplings to grow sufficiently fast out of the disturbance zone so as to reach fire proof (3m), frost proof (4m), and herbivore proof (10m) heights (Stevens et al., 2018).

The chances of successful escape are enabled by low grass biomass, which facilitates faster growth rates (Stevens et al., 2018; Vadigi & Ward, 2013), as well as a low frequency of top‐kill‐inducing disturbance events (i.e., frost, fire and herbivory) (Stevens et al., 2018; Whitecross et al., 2012). When saplings do experience top‐kill, there is a low mortality among the saplings and they rapidly resprout in the following growing season. However, the repeated top‐kill events result in the disturbed trees experiencing shifts in height and architecture (Caughley, 1976; Kennedy & Potgieter, 2003; Stevens et al., 2018; Styles & Skinner, 2000; Whitecross et al., 2012). This occurs as the resprouting saplings coppice and has a strong tendency to regrow as multi‐stemmed shrubs, which are short and seldom grow above 4m (Kennedy & Potgieter, 2003; Stevens et al., 2018; Styles & Skinner, 2000; Whitecross et al., 2012).

#### 2.3.1 Top‐kill by fire and herbivory

Mopane saplings are primarily damaged by small‐ and medium‐sized browsers (Moe et al., 2009). Mopane twigs and leaves are palatable particularly when they are young and as a result mopane faces regular browsing events (Smit, 2001; Styles & Skinner, 2000). Similar to seedlings, browsed saplings resprout with more stems, lateral shoots, and branches effectively converting the sapling from a single stem to a multi‐stemmed growth form (Archibald et al *in prep,* Rooke et al., 2004). Although elephant impact is low at this stage, the impacts of elephant trampling could be important; however, it has not been investigated for this species (Poulsen et al., 2018). An intermediate level of defoliation causes saplings to increase the synthesis of condensed tannins, but this concentration is lowered when defoliation is intense (Kohi et al., 2010). This creates a positive feedback between browsers and intensively browsed saplings (Kohi et al., 2010) promoting regular revisits by browsers.

Fire also causes significant shifts in the architecture of mopane and can convert single‐stemmed mopane saplings into multi‐stemmed shrubs (Mlambo & Mapaure, 2006) where regrowth after top‐kill from fire is characterized by stands of short multi‐stemmed shrubs (Mlambo & Mapaure, 2006). This architecture prevents the saplings from moving into the next size category, and even if a disturbance‐free period occurs as multi‐stemmed saplings lose apical dominance, and face a trade‐off with height gain where single‐stemmed saplings can divert resources to maximizing rates of vertical extension and shrubs cannot (Mlambo & Mapaure, 2006).

### 2.4 Adult plants

#### 2.4.1 Tree mortality

There are few, if any clear direct limits to the survival of adult mopane trees. Drought impacts on mopane are low as they have a high tolerance to heat, and drought due to features that reduce water loss and water stress (Makhado, Mapaure, et al., 2014; O’Keefe et al., 2016) can continue to photosynthesize with little ill‐effect under these conditions (Midgley et al., 2004; O’Keefe et al., 2016; Stevens et al., 2016). As a result, droughts appear to only cause localized patch dieback and limited mortality where trees taller than 5m are most affected (Macgregor & O’Connor, 2002; Viljoen, 1995).

Top‐kill of adult mopane seldom causes mortality (Smith & Shah‐Smith, 1999). Records of mortality associated with herbivory (mostly elephant damage) range from 0.5% for coppiced trees and 0% – 3% for tall trees under low elephant densities (Lewis, 1991) to 12% under high elephant densities (Ben‐Shahar, 1996a). Fire‐associated mortality also appears low with a 1% mortality rate recorded in Chobe National Park (Ben‐Shahar, 1996a; Lewis, 1991). Although frost is presumed to be a primary agent in limiting the range of mopane (Burke, 2006; Henning & White, 1974), there are very limited records of frost‐induced mortality in mopane (Stevens et al., 2018). Instead, mopane is physiologically tolerant to freeze events with frost only causing top‐kill in shrub mopane shorter than 4m in height (O’Keefe et al., 2016; Whitecross et al., 2012).

#### 2.4.2 Top‐kill in adult trees

##### Herbivory

Adult mopane coppices readily after heavy browsing. The most significant herbivory impact to adult trees is from heavy elephant damage, which is recorded widely across its distribution range (Ben‐Shahar, 1996a, 1996b; Hrabar, 2005; Kennedy & Potgieter, 2003; Macgregor & O’Connor, 2002; Mapaure & Mhlanga, 1998, 2000; Smallie & O’Connor, 2000; Styles & Skinner, 2000; Van Wyk & Fairall, 1969). Elephant utilization takes a variety of forms; from leaf stripping to whole twigs and smaller branches being removed, to bark stripping and ring barking of the main stem (Lewis, 1991; Smallie & O’Connor, 2000). Trees with a girth up to 130cm are vulnerable to main stem snapping, and trees with a girth up to 190cm can be pushed over (Caughley, 1976). Elephant browsing most significantly changes the structure of the tree population and can result in the removal of between 14 and 48% of biomass with shrubs experiencing significantly more biomass removal than tall trees (Ben‐Shahar, 1993). High levels of herbivory result in a reduced stem diameter growth rate, lower leaf production and lower mean individual leaf masses (Kohi et al., 2010). Elephants have strong preference for coppicing trees that have a prevalence of branches with diameters between 1 and 1.7 cm (Ben‐Shahar, 1996a, 1996b; Kohi et al., 2010; Lewis, 1991; Mapaure, 2011; Smallie & O’Connor, 2000). Thus, regular browsed coppicing trees are more likely to be regularly visited (Kohi et al., 2010; Smallie & O’Connor, 2000). The progressive herbivory over time results in the shift of a population from tall single‐stemmed trees to intensively coppicing multi‐stemmed short shrub stands (Caughley, 1976).

Mopane is also browsed by a range of other mammals including goats, eland, impala, kudu, steenbok, gray duiker, giraffe and even wildebeest and zebra (Donaldson, 1979; Styles & Skinner, 1997; Timberlake, 1995) particularly during the dry season as the leaves remain green long into the dry season (Dekker & Smit, 1996; Hooimeijer et al., 2005; Stevens et al., 2016) and retain their nutritional value when fallen on the ground or are in stages of senescence (Kelly & Walker, 1976). However, herbivory by these mammals on adult trees has relatively low impacts.

##### Mopane caterpillar

Mopane trees have a unique relationship with the larvae of the mopane moth (*Gonimbrasia belina*). The caterpillars are folivores that specialize on mopane leaves (Picker, 2012). They usually hatch in November and feed on mopane leaves for about 6 weeks (Gaston et al., 1997). In some years, a second generation may hatch in February or March (Harabar et al., 2009). The abundance of these caterpillars can be so high to the extent that mopane can be completely stripped of their leaves (Hrabar et al., 2009; Timberlake, 1995). Defoliation by these caterpillars does not cause mortality or top‐kill, and following defoliation tree canopies can undergo a complete reflush of their leaves. However, the regrowth results in a decrease in the length of shoot (−50%) and leaf size (−80%) and decreased chemical defense compounds in the leaves (Hrabar et al., 2009).

##### Human utilization

Mopane woodlands provide important ecosystem services to human communities living within their range (Woollen et al., 2016). They are heavily utilized for building material and charcoal production and face a heavy harvesting pressure. Following regular harvesting, mopane responds with vigorous coppice regrowth with an average of 5.3 (range 1 to 27) coppice shoots per stump following main stem harvesting (Mapaure, 2011), with harvesting pressure shifting woodland structure toward smaller multi‐stemmed shrubs.

##### Fire

Fire causes significant changes in the mopane stand structure. Following fire, mopane resprouts producing an average of six resprouted shoots per shrub four months after a fire (Mlambo & Mapaure, 2006), which can increase to up to eight one year after the fire (Kennedy & Potgieter, 2003) with the number of resprouts independent on shrub size, indicating that the number of activated dormant buds is size independent. Hence, frequently burnt stands of mopane are significantly shorter and experience a 100% increase in the proportion of shrubs and a 3‐fold increase in the mean stem density as a result of increased coppicing (Gandiwa & Kativu, 2009; Higgins et al., 2007; Kennedy & Potgieter, 2003; Nefabas & Gambiza, 2007). Where fire frequency is high, mean tree height is half that of trees in areas that are seldom burnt.

#### 2.4.3 Physiognomy and stand structure of mopane

Mopane is found on a range of soils from heavy, calcareous sodic soils to sandstone, granites, basalts, Kalahari sands, alluvium, and shale (Dye & Walker, 1980; Makhado, Mapaure, et al., 2014; Mapaure, 1994; O’Connor, 1992). As it occurs on a wide variety of substrates, soil characteristics alone are unlikely to predict the distribution of mopane. However, edaphic factors can shape mopane physiognomy, that is, shrub versus tall tree forms. Edaphic control of mopane physiognomy is strongest in discrete patches consisting of heavy clays and on sodic soils (Dye & Walker, 1980) where shrub mopane (sometimes referred to as bonsai mopane) is common. Tall mopane (also referred to as cathedral mopane) occurs on relatively more permeable soils (Dye & Walker, 1980; Lewis, 1991; Mantlana, 2002).

However, short and tall forms of mopane are also widespread where they are not a product of edaphic processes. Top‐kill that results in resprouting cause mopane stand structure to shift from that of tall single or two stemmed trees to stands short multi‐stemmed shrubs. As the mopane tree is incapable of self‐thinning, once top‐killed, the resprouting shrub does not develop a single leading shoot that becomes a tree but instead remains a multi‐stemmed shrub where growth is diffused across lateral stems. This fundamental modification of stand structure would be of academic interest except the seeding ability of mopane is a function of tree height. With progressive elimination of tall single‐stemmed trees, the source of mopane seeds is also eliminated (Caughley, 1976) and frequent top‐kill can result in large areas of the mopane dominated landscape becoming functionally sterile.

## 3 WHAT SHAPES MOPANE’S DISTRIBUTION RANGE?

**FIGURE 1:**
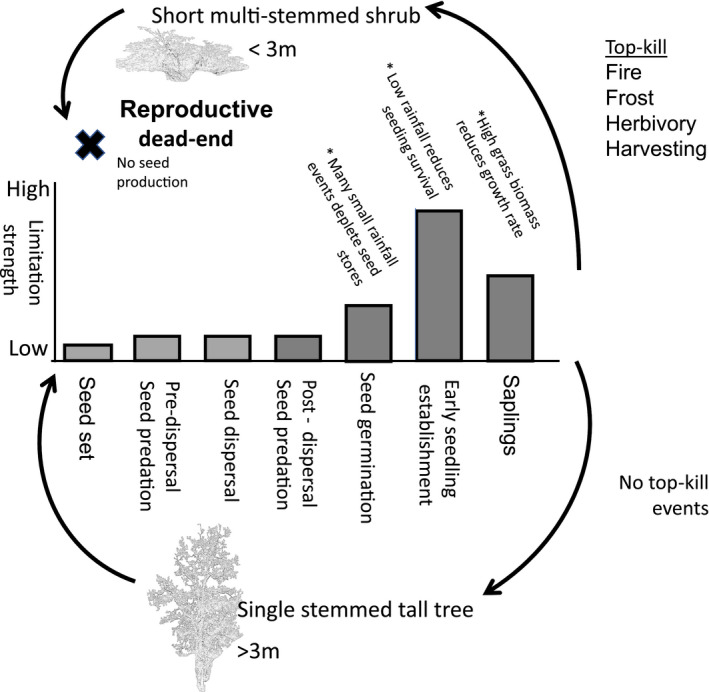
The life cycle of *Colophospermum mopane*. The bar graph denotes the relative strength of the demographic hurdle at each life‐history stage. When seedlings become successfully established the critically limiting step is top‐kill, the legacy of which shapes their future trajectory of reproduction. If a seedling, sapling, or adult plant experiences significant top‐kill its regrowth results in coppicing causing a shift from a single‐stemmed tree to a multi‐stemmed shrub. The ability of a short multi‐stemmed shrub to produce seeds is severely impaired thus resulting in shrub stands that are effectively sterile

Multiple population bottlenecks occur during the lifecycle of this plant (Figure 1 *for overview*). The first bottleneck occurs in the seed bank. Small rainfall events are sufficient to facilitate seed germination but insufficient to ensure the subsequent survival of the germinating seedling (Stevens, Seal, et al., 2014) and can, therefore, result in the depletion of a whole seasons seed supply (Figure 3). I propose this process may have the strongest limit where multiple small rainfall events are common, especially in the early growing season. The next limiting step is that of early seedling survival. The newly germinated seedling requires sufficient follow‐up rainfall to ensure the extension of the growing radicle out of the evaporation zone in the soil which on average appears to result in strongly episodic recruitment every 3 years (Stevens, Seal, et al., 2014). I propose that this limitation is strongest in the arid savannas of this species distribution range. The third limit is shaped by the plant's architecture across all demographic stages, with this critically limiting step shaped by the probability of top‐kill and legacy of disturbance (Figure 1). Mopane distribution is best predicted by its ability to reach top‐kill proof heights in areas where the time period between disturbances is greater than the time it takes to reach escape height (Stevens et al., 2018). Here, if mopane is exposed to top‐kill, it is unlikely to experience mortality and instead will resprout as a multi‐stemmed coppicing shrub. The legacy of top‐kill means that it is unlikely that the resprouting multi‐stemmed shrub will ever become a successfully recruiting adult tree as the probability of producing seeds in the short multi‐stemmed shrub form is extremely low. Patches of mopane facing stand wide fire, frost, intensive herbivory and harvesting are, therefore, unlikely be able to produce seeds again resulting in many large tracts of land containing this species being effectively sterile.

**FIGURE 2:**
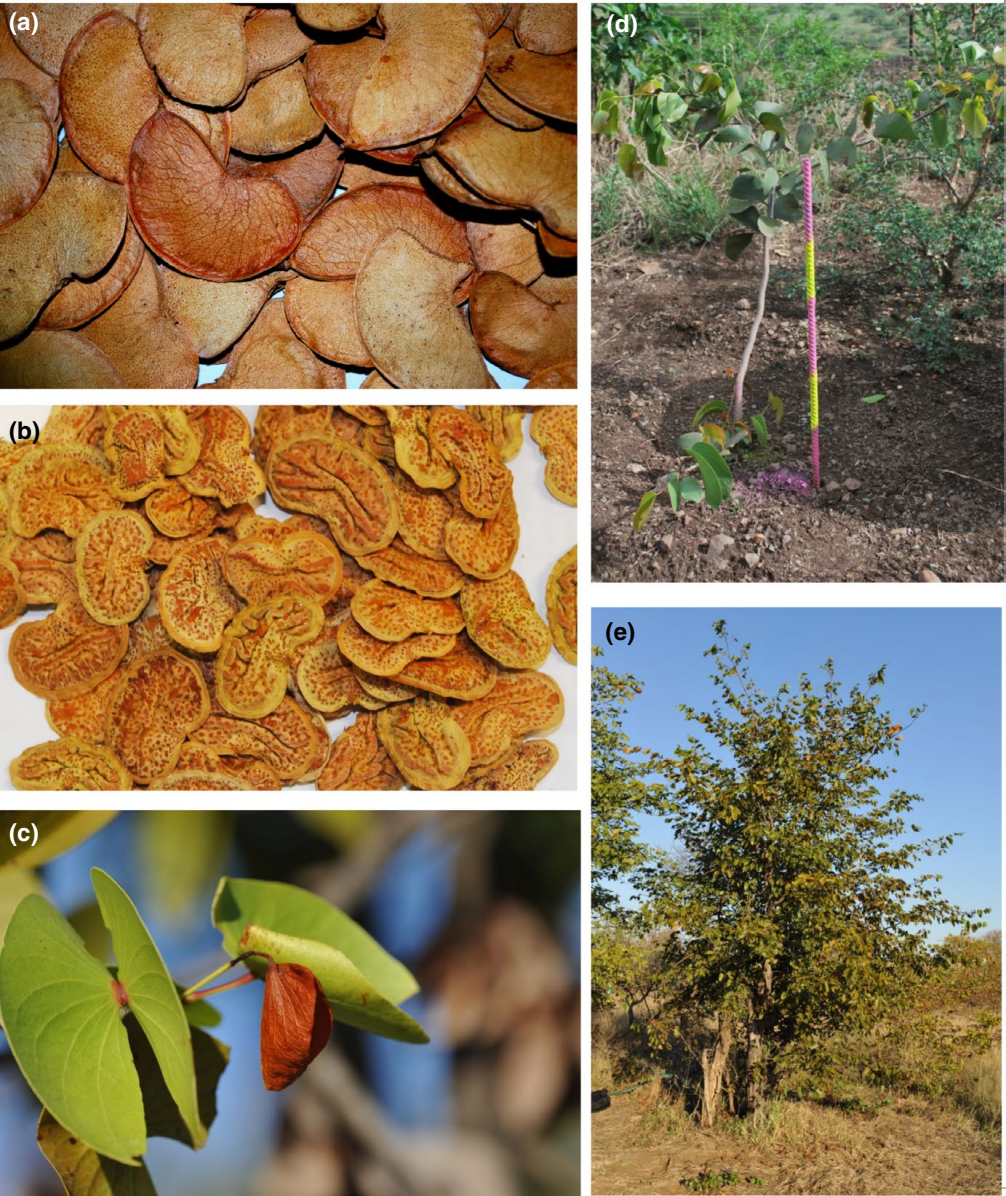
a) Mopane seed pods (40‐50mm) and b) seeds removed from seed pods (30‐40mm). c) Butterfly shaped leaves of mopane, d) a 2‐year‐old sapling (each yellow/pink interval indicates 10cm), and e) an adult tree (8m tall)

**FIGURE 3:**
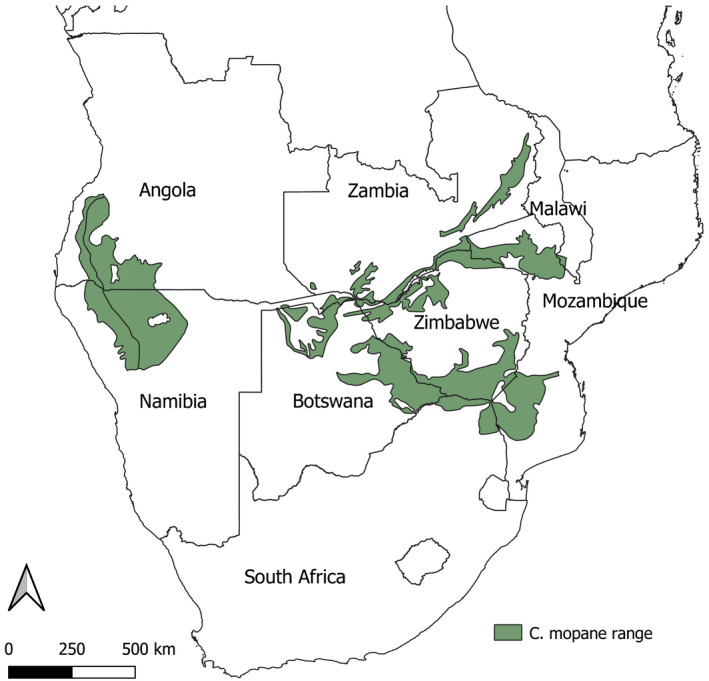
Distribution range of *Colophospermum mopane*. Data extracted from White (1983), Vegetation of Africa

I propose, therefore, that range limits coincide with areas where the probability of top‐kill increases. As fire frequency increases with increasing rainfall, the probability of top‐kill by fire is high and may explain the correlation with rainfall (>800mm) observed in species distribution models (Henning & White, 1974; Makhado, Mapaure, et al., 2014; Stevens, Swemmer, et al., 2014). Similarly, the correlation with frost and range edges may explain why frost is considered to result in direct range limits of this plant (Burke, 2006; Stevens, Swemmer, et al., 2014). The probability of herbivory and harvesting are likely landscape dependent and the impacts of these disturbances on the distribution of this species can be predicted by local landscape features like distance to permanent water or walking distance to nearest village. Thus, I propose that whilst experimental work shows that mopane can grow and even thrive well outside its proposed climatic range limits (Stevens et al., 2018) it is the probability of the reduction in population seed production that ultimately limits this species. Therefore, I predict that at the range edge of this species the probability of top‐kill causing disturbance is highest and results in an increase in the proportion of “effectively sterile trees” in the landscape.

## 4 CONCLUSION

In this review, I demonstrate that multiple and often interactive processes limit the success of mopane, the strength of which varies between demographic stages. I highlight how important top‐kill‐inducing disturbance is in shaping the success of this species and show that the legacy of disturbance can have consequences for the future success of plants. This can inform us which management interventions can cause range changes due to changes in disturbances and will facilitate practical management guidelines for conservation areas and rangelands to ensure continued reproductive success.

Insights from this can serve as a framework to understand the range limits of other dominant savanna species, particularly those that require a height threshold to produce seeds (Midgley et al., 2020; Wright et al., 2005). This approach points to mechanistic misunderstandings that can occur when using climate‐based species distribution models as a tool to identify range limits in disturbance driven systems, something that is of increasing importance given our need to accurately predict the current and future ranges of plants under global change. Furthermore, it suggests that where global change is causing fundamental changes to the disturbance regimes, species ranges can be altered.

## CONFLICT OF INTEREST

None declared.

## AUTHOR CONTRIBUTION

**Nicola Stevens:** Conceptualization (lead); Data curation (lead); Investigation (lead); Methodology (lead); Project administration (lead); Resources (lead); Visualization (lead); Writing‐original draft (lead); Writing‐review & editing (lead).

## Data Availability

This is a review and uses no new data.
